# Fast Bowler’s knee – anteromedial articular impingement

**DOI:** 10.1186/s40634-020-00237-7

**Published:** 2020-04-08

**Authors:** Michael J. Reid, Avinash Alva, Simon M. Thompson, Ranju T. Dhawan, Mary H. Jones, Andy M. Williams

**Affiliations:** 1 Cairns Hospital, 165 The Esplanade, Cairns City, QLD 4870 Australia; 2 James Cook University, 1 James Cook Dr, Townsville City, QLD 4811 Australia; 3 Fortius Clinic, 17 Fitzhardinge Street, London, W1H 6EQ UK; 4 Chelsea and Westminster NHS Trust, 369 Fulham Road, London, SW10 9NH UK; 5 Radiology Department, Imperial College NHS Healthcare Trust, The Bays South Wharf Road St Mary’s Hospital, London, W2 1NY UK; 6 Hybrid Imaging, Wellington Hospital, Wellington Place, London, NW8 9PY UK

**Keywords:** Articular impingement, Fast bowling, Cricket

## Abstract

**Purpose:**

To describe a series of impingement lesions found on the anterior aspect of the medial femoral condyle in international cricketers.

**Methods:**

Seven international level fast bowlers presented to our clinic with knee pain in the lead leg between 2005 and 2013. The mean age of the patients was 26.7 years (20–29 years). In all patients a careful history and examination was undertaken followed by appropriate investigations. Conservative management and arthroscopic surgery were performed on these cases. We aimed for a pain free quiet knee with resolved oedema on MRI and return to sport.

**Results:**

MRI images showed oedema in the medial femoral condyle in all patients and 4 patients also had associated cartilage loss. These 4 patients underwent arthroscopic surgery whereas the other 3 were less symptomatic and were managed conservatively. All patients returned to international cricket at an average of 6 months in the non-operative group and 8 months in the operative group.

**Conclusion:**

Anterior impingement of the anteromedial femoral condyle can be a potentially serious lesion in the fast bowler. A strong index of suspicion regarding this lesion has to be exercised when a fast bowler attends with knee pain and effusion.

## Introduction

The popularity of the game of cricket has increased since the introduction of limited over cricket (20 and 50 over format) and this has led to a significant expansion in the number of matches played, particularly at international level [1]. Fast bowlers have been shown to have the greatest workload compared to other cricketers, covering between 20 and 80% more distance and this is 2–7 times more likely to be at a higher intensity [2]. It is thought that there is a correlation between bowling workload and injury risk, suggesting that injuries may be related to overuse. It has been estimated that a fast bowler may bowl as many as 300 deliveries during a 4 or 5 day game [1]. With the ground reaction force during the front foot contact phase of a delivery being quoted as between 3 and 9 times bodyweight there is considerable repeated loading of the limb [3]. It was found in the front leg in the ‘delivery-stride’ (i.e. left leg for a right arm bowler), which is the final pace, during which the ball is released. We report on a series of seven elite level international fast bowlers who presented with a similar injury pattern to the anterior aspect of the medial femoral condyle of the knee. We describe the presentation, investigation and treatment of this lesion and discuss the possible etiology. This injury pattern has not previously been reported in the literature.

## Findings

Seven international level cricket fast bowlers presented to our clinic with knee pain in the lead leg (front landing leg during bowling) between 2005 and 2013. The mean age of the patients was 26.7 years (20–29 years). In all patients a careful history and examination was undertaken followed by appropriate investigations. The main complaint was that of anteromedial knee pain which was restricting them from bowling. The duration of symptoms varied between approximately 6 weeks and 2 years. One case presented after 2 weeks but closer questioning revealed a much longer history of intermittent pain.The anteromedial knee pain was associated with a minor fixed flexion deformity in three of the patients but otherwise there was full range of motion. Muscle atrophy was common in the affected limb and all patients had an effusion at the time of presentation. All patients underwent MRI and Spect CT. We recorded outcomes after treatment. The expected outcomes of treatment was a pain free quiet knee with complete absence of oedema on MRI and return to sport to same level at the time of injury.

**Fig. 1:**
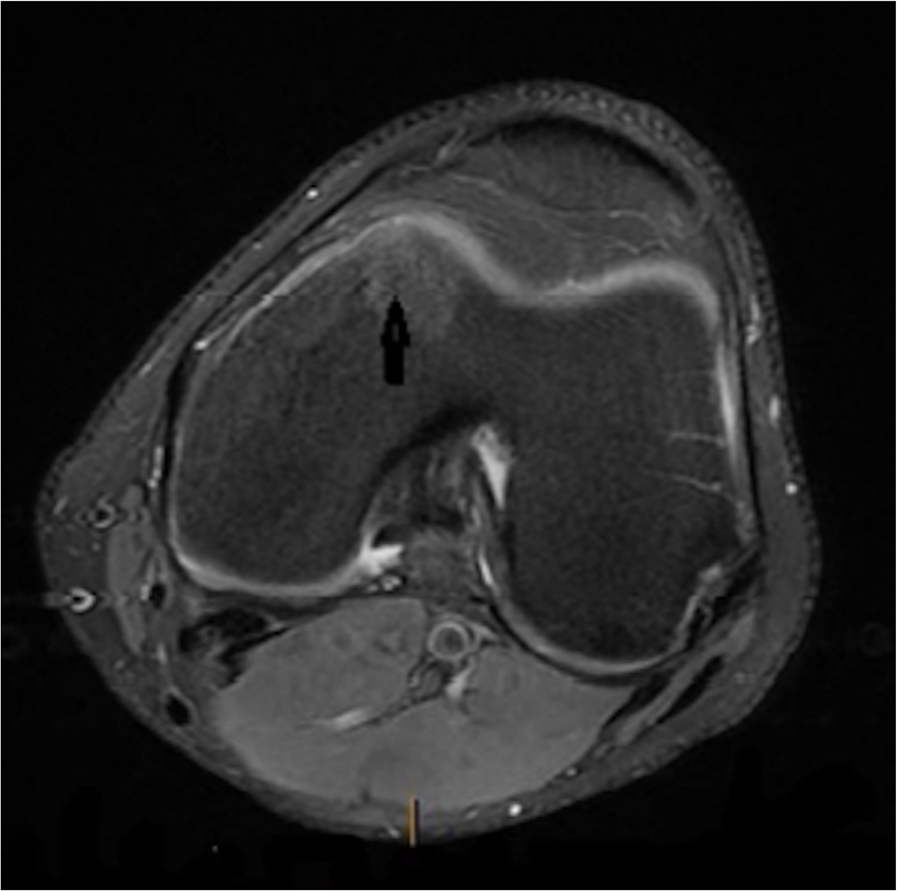
Axial MRI of the left knee showing oedema in the medial femoral condyle

**Fig. 2:**
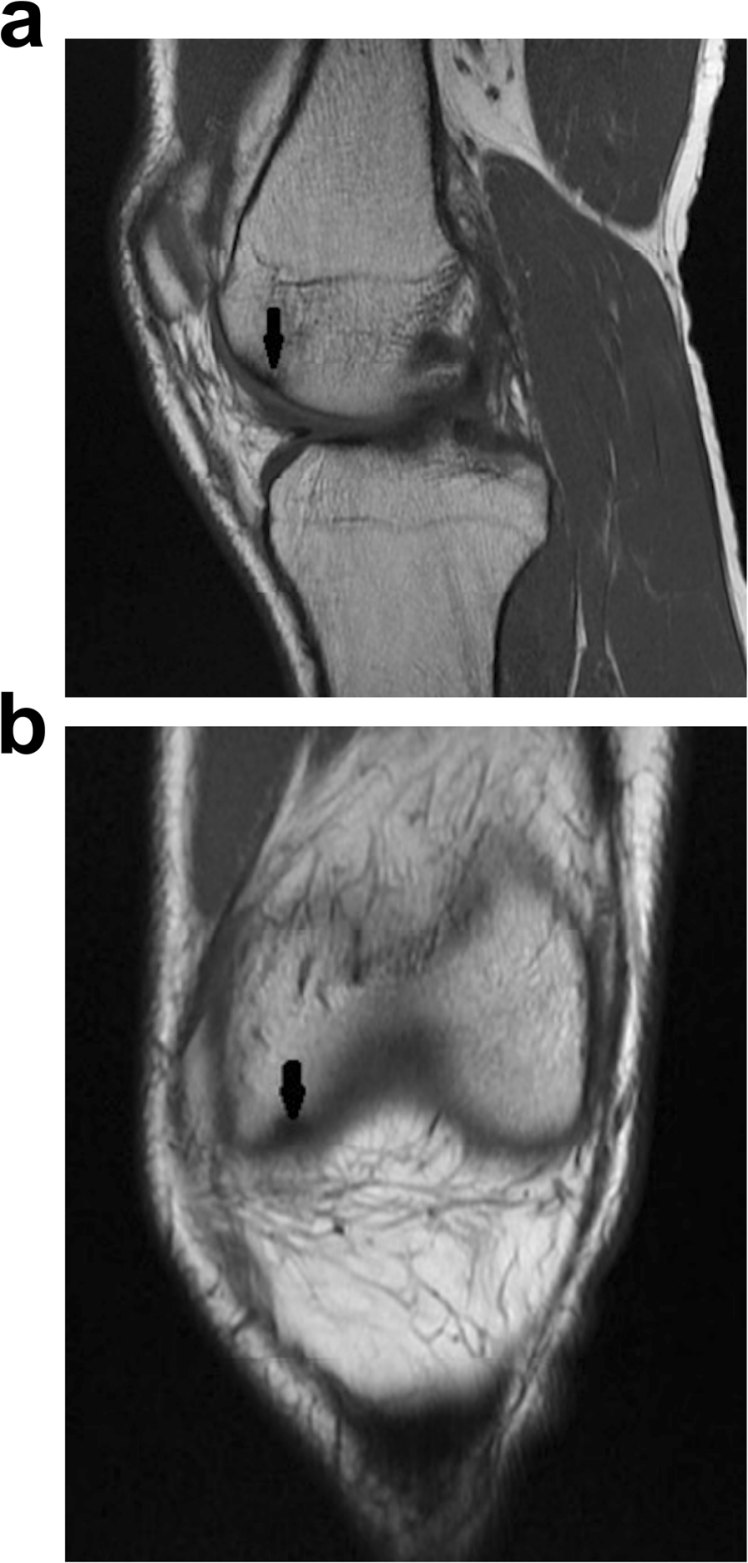
Left Knee T1 MRI showing subtle abnormality of subchondral bone in the same patient. **a** Sagittal view, **b** Coronal view

**Fig. 3:**
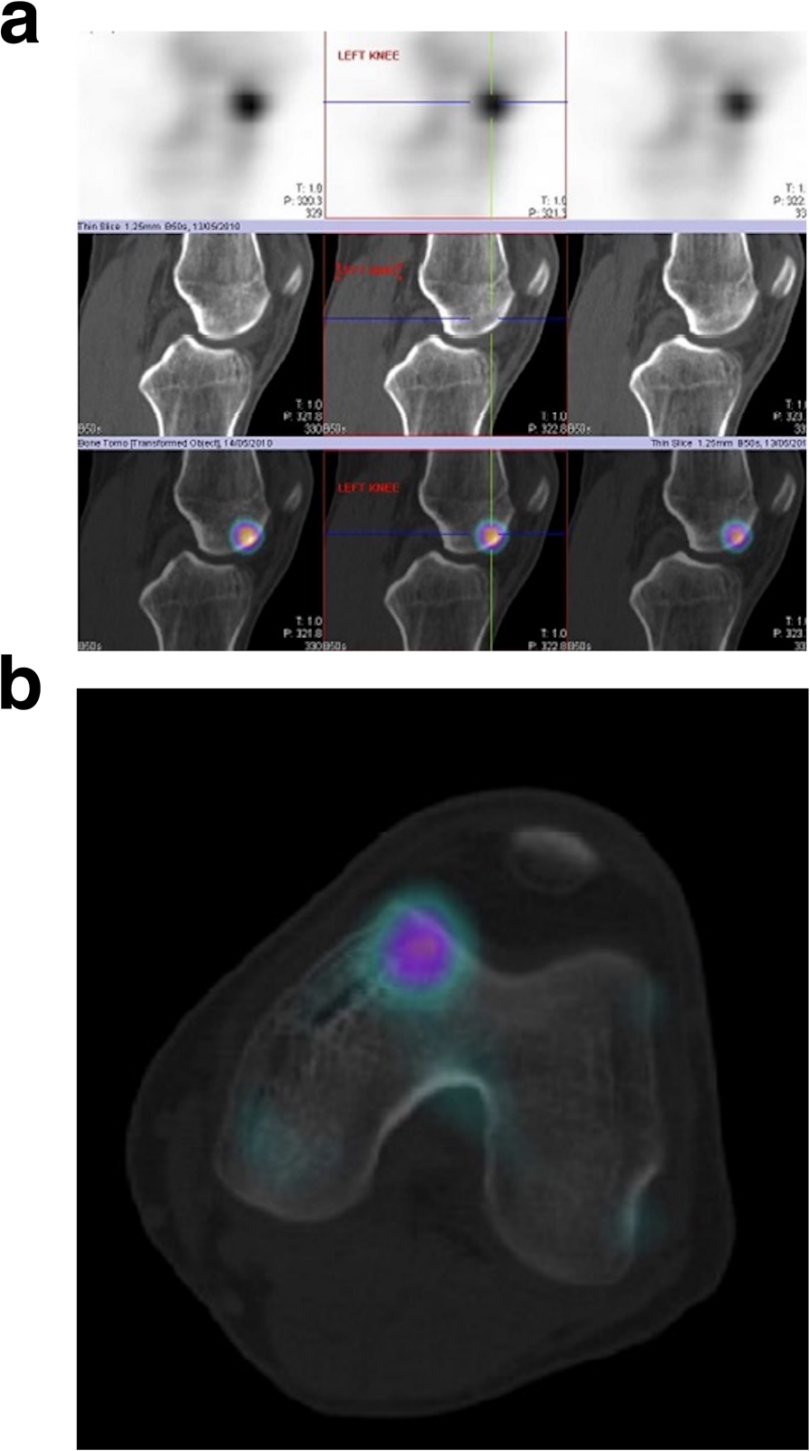
**a** Sagittal SPECT-CT scan slices of the same patient demonstrating obvious abnormality with focally increased tracer activity pertaining to the sub-chondral aspect of the medial femoral condyle. **b** Tranaxial SPECT-CT slice of the left knee highlighting the lesion on the medial femoral condyle

**Fig. 4:**
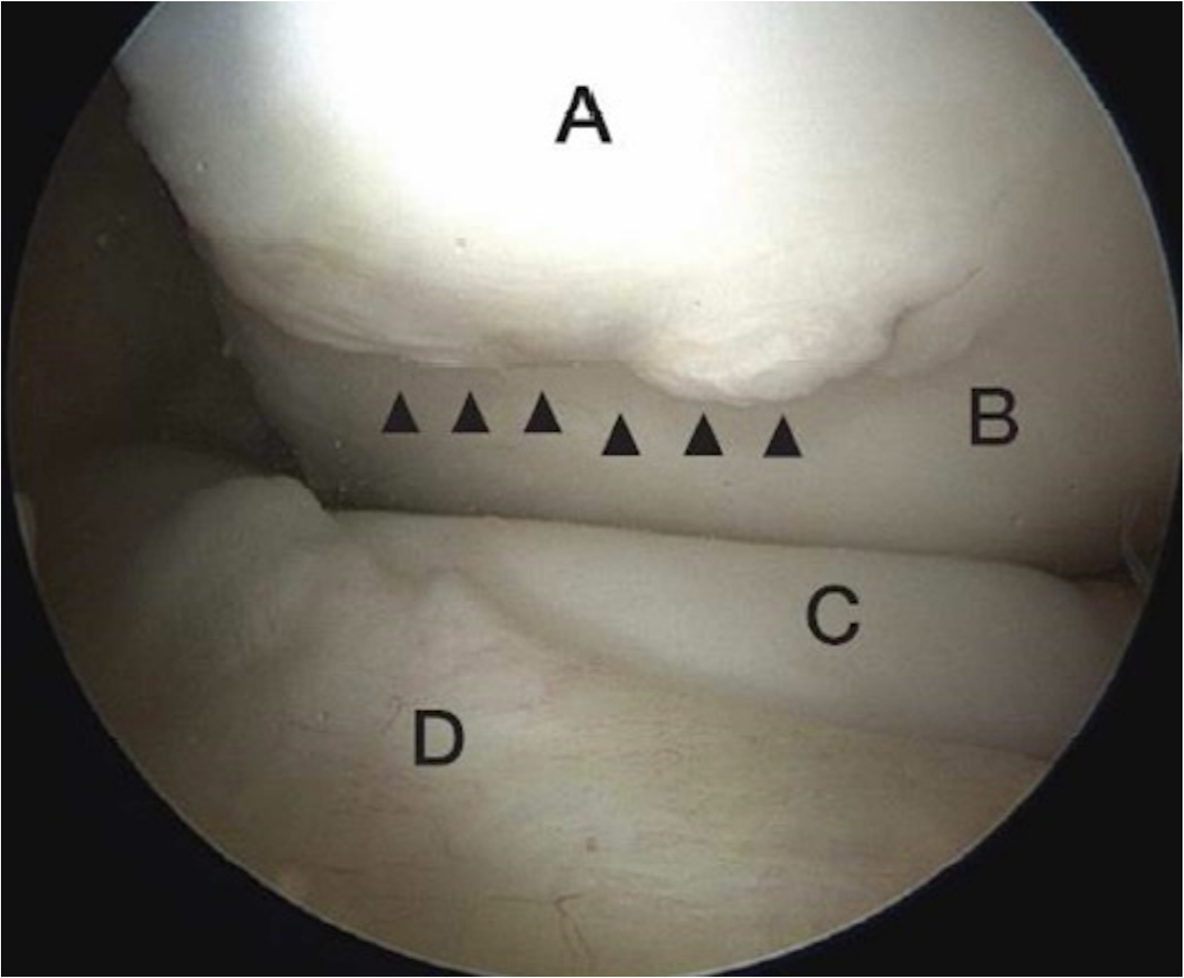
View of anterior medial compartment showing impingement lesion of the medial femoral condyle. **a** Impingement Lesion, **b** Normal Femur, **c** Normal Anterior Tibia, **d** Anterior Horn of Medial Meniscus

All patients underwent an MRI scan in which the classical appearance of oedema within the medial femoral condyle (Fig. 1) was noted. In 4 patients who were more symptomatic, there was associated articular cartilage loss noted on the MRI. In the less symptomatic patients, the MRI abnormality was not especially notable (Fig. 2a, b and Fig. 3a, b) but the injury was more conspicuously also identified on Bone HDP (hydroxydiphosphonate) SPECT-CT scan. The Bone HDP SPECT-CT scan (a combination of 3D SPECT slices of the Tc99m HDP bone scan with CT overlay) was especially useful in highlighting the osteochondral activity responsible for symptoms, whilst the template CT demonstrated structural / anatomical characteristics. (Fig. 4).

Four of the cases who were more symptomatic and showed chondral damage on MRI were treated with arthroscopy of the knee in the form of excision of delaminated articular cartilage and microfracture of the lesion. The lesion on the medial femoral condyle was always sited where the tibia / anterior horn of meniscus would impinge in hyperextension. In the cases requiring arthroscopic surgery with extension of the knee this phenomenon could be confirmed visually. In Fig. 4 note the prominence of the anterior upslope of the tibia, which abuts the lesion in full extension. As can be seen the anterior horn of the medial meniscus translates anteriorly in extension to avoid impingement in the joint. Average size of chondral damage on arthroscopy was 1.5 by 1 cms.

Three patients found to have oedema alone on the MRI and with less severe symptoms were managed non-operatively. Conservative management of these patients involved anti-inflammatory medications, relative rest with avoidance of impact loading of the knee and oedema management. Once the effusion had subsided a strengthening and conditioning programme aimed at the trunk, pelvis and both lower limbs was commenced and progressed according to their symptoms. Following surgery there was a 6–8 week non weight-bearing period during which range of motion exercises were encouraged. This was followed by the same strength and conditioning programme as those treated conservatively. A careful, graduated return to bowling ensued and a strict maintenance strategy of strengthening and conditioning was recommended. A return to playing duties was achieved once the patient had a pain free, quiet knee and appropriate neuromuscular control to protect the knee.

Final MRIs of all patients confirmed that the oedema had completely resolved on MRI. The knee was pain free and quiet at final clinical follow-up. All of them returned to International cricket at an average of 6 months in the non-operative group and 8 months in the operative group.

## Discussion and review of literature

To achieve bowling speeds of upto 90 miles per hour fast bowlers generate large ground reaction forces on the leading leg at the point of delivery [3]. Peak ground reaction forces occur within the first 50 m/s of front foot contact in the delivery stride and can reach 9 times body weight. There is a suggestion that these forces are greatest in players with the most extension at the point of front foot contact and in those that flex the least between first foot contact and ball release meaning the forces are absorbed by the knee joint rather than the musculature [3]. These greater forces through the joint, repeated with every delivery could result in microtrauma. We believe, therefore, that the pathological process causing the lesion presented here is one of repetitive and accumulative overload impingement of the anterior medial femoral condyle during the delivery stride foot strike by the lead leg [4]. Impaction fracture of the medial femoral condyle have also been reported from hyperextension injuries in football [5]. A systematic review on chondral injuries in soccer players by Andrade et al. showed the most common location of the lesion was the medial femoral condyle (51% of the 217 lesions).

In 2005 the senior author, Williams A et al. published on knee kinematics as studied by ‘dynamic’ MRI [6]. Some of the findings from this study and others describe the events in hyperextension that occur regarding joint contact [7, 8]. In the sagittal plane extension causes anterior displacement of both meniscal anterior horns. On the lateral side of the joint there is good clearance of the articulating surfaces as in the sagittal plane the tibial surface falls away inferiorly. There is also the sulcus terminalis to accept the anterior horn of lateral meniscus. In contrast, on the medial side of the knee, whilst the anterior horn of the medial meniscus moves out of the way, there is compression between the anterior portion of the medial femoral condyle and the anterior up slope on the medial tibia in the sagittal plane [9]. This pattern of motion means that hyperextension results in compression of the anteromedial compartment of the knee. In the situation of a fast bowler the injury is similar in site and occurs through attritional overload.

In early cases before structural damage has occurred, relative rest can allow the lesion to heal as was achieved in our series of cases. However, once structural damage is established surgical intervention is may be warranted. The nature of this injury makes it potentially career ending although that has not been the experience in our series.

## Conclusion

Anterior impingement of the anteromedial femoral condyle can be a potentially serious lesion in the fast bowler. Left untreated this can progress to a career ending situation. It is most likely to occur in elite players due to their packed match schedules and it may be related to the repetitive loading of the leading knee in extension. A strong index of suspicion regarding this lesion has to be exercised when a fast bowler attends with pain and especially an effusion. Articular damage confirmed on scans are best treated with arthroscopic debridement of delaminated cartilage and microfracture. In our experience, fast bowlers with such knee conditions can successfully return to sport following treatment. Modifiable intrinsic factors, such as balance and proprioception should be included in preparticipatory screening and injury prevention. Improving sagittal-plane landing mechanics is important in reducing harmful magnitudes and directions of impact forces on the knee. A constant moderate bowling workload reduces the risk of injury in these players. This paper does have limitations in the form of a small patient group. However, we recommend that medical staff need to be aware of this lesion in order that prompt intervention may allow healing of the lesion.

## Data Availability

Not relevant.

## Abbreviations

GRF: Ground reaction force
MFC: Medial femoral condyle
MRI: Magnetic resonance imaging
U-19: Under 19 years
